# DEMETER plant DNA demethylase induces antiviral response by interferon signalling in animal cells

**DOI:** 10.1038/s41598-017-08827-9

**Published:** 2017-08-22

**Authors:** Young Geun Mok, Ki Young Choi, Seung Hwan Hong, Jin Hoe Huh

**Affiliations:** 1 Interdisciplinary Program in Agricultural Genomics, Department of Plant Science, Plant Genomics and Breeding Institute, and Research Institute of Agriculture and Life Sciences, Seoul National University, Seoul, 08826 Korea; 2 School of Biological Sciences, Seoul National University, Seoul, 08826 Korea

## Abstract

DNA methylation is a prominent epigenetic modification in plants and animals regulated by similar mechanisms but the process of DNA demethylation is profoundly different. Unlike vertebrates that require a series of enzymatic conversions of 5-methylcytosine (5mC) into other bases for DNA demethylation, plants utilize the DEMETER (DME) family of 5mC DNA glycosylases to catalyze a direct removal of 5mC from DNA. Here we introduced *Arabidopsis* DME into human HEK-293T cells to allow direct 5mC excision, and observed that direct DNA demethylation activity was successfully implemented by DME expression. In addition, DME induced diverse cellular responses such as cell proliferation inhibition, cell cycle dysregulation and S phase arrest. Microarray and methylome analyses revealed that DME upregulated a number of genes including cell cycle components, heat shock proteins, and notably, various interferon-stimulated genes. Moreover, DME-mediated DNA demethylation activated endogenous repeat elements, which are likely to form dsRNAs as viral mimics and eventually trigger interferon cascades to establish the antiviral state. This work demonstrates that plant DNA demethylase catalyzes DNA demethylation with a bypass of initial base conversion steps, and the interferon signaling plays a pivotal role to alleviate genotoxic stresses associated with DME-induced DNA demethylation in mammalian cells.

## Introduction

DNA methylation has a variety of functions in many cellular processes such as transcriptional regulation, differentiation, gene imprinting and transposable element silencing^1–3^. It is believed that plants and animals have evolved similar mechanisms of DNA methylation in terms of overall processes and the enzymes that catalyse the transfer of a methyl group onto a cytosine base to produce 5-methylcytosine (5mC), which is presumably the most stable and universal epigenetic mark in eukaryotes.

DNA methylation can be dynamically regulated in response to developmental cues, for which the process of DNA demethylation plays a critical role. DNA demethylation takes place in a passive or active mode. Passive DNA demethylation is replication-dependent, and the inhibition of DNA methyltransferase (DNMT) results in a gradual decrease in the genome-wide DNA methylation level over cell divisions. In contrast, active DNA demethylation is replication-independent, and DNA methylation is enzymatically removed without cell division. The most fundamental difference between the plant and animal DNA demethylation pathways probably lies at the initial step of active DNA demethylation, in which completely different enzymatic activities are engaged.

Plants utilize DEMETER (DME)/REPRESSOR OF SILENCING 1 (ROS1) DNA glycosylase family proteins to specifically recognize and excise 5mC from DNA^4–6^. Seeds are the products of sexual reproduction in flowering plants consisting of seed coat, embryo and endosperm, and DME plays an important role for seed development^4, 7^. In *Arabidopsis* DME is primarily expressed in the central cell of the female gametophyte, the progenitor cell of endosperm that nourishes the embryo. DME removes DNA methylation at discrete loci in the central cell, and such changes in DNA methylation are mitotically inherited to dividing endosperm cells after fertilization^8^. Some DME targets include *MEA*, *FIS2* and *FWA* genes, which are imprinted in endosperm where only the maternal alleles are expressed^4, 9, 10^. In parallel, DME is also expressed in vegetative cells of pollen, the male gametophyte^11^. It is believed that DME induces demethylation of many transposable elements (TEs) in the central cell and vegetative cells producing small RNAs, which are then likely to translocate to nearby gamete cells such as an egg and sperm in the female and male gametophytes, respectively, in order to reinforce methylation and silencing of corresponding TEs *in trans* ^12^.

In animals, the ten-eleven translocation (TET) family of proteins catalyse successive conversions of 5mC into 5-hydroxymethylcytosine (5hmC) and higher oxidative derivatives 5-formylcytosine (5fC) and 5-carboxycytosine (5caC) prior to base excision by a T:G mismatch-specific thymine DNA glycosylase (TDG)^13–17^. In addition, deamination of 5mC by activation-induced cytidine deaminase (AID)/apolipoprotein B mRNA editing enzyme, catalytic polypeptide-like (APOBEC) family proteins are also proposed to participate in the base conversion process^18^. Therefore, active DNA demethylation in animals requires additional steps at the initial stage compared to the plant demethylation pathway.

In this study, we introduced *Arabidopsis* DME DNA demethylase into HEK-293T cells and investigated the consequence of direct 5mC excision in animal cells. We found that DME expression inhibits cell proliferation rate associated with DNA damage and S phase arrest. Remarkably, direct excision of 5mC triggered interferon cascades using TE-derived dsRNAs as viral mimics, demonstrating that active DNA demethylation is associated with antiviral response in animal cells.

## Results

### Expression of *Arabidopsis* DME DNA demethylase confers direct 5mC excision activity to mammalian cells

**Figure 1:**
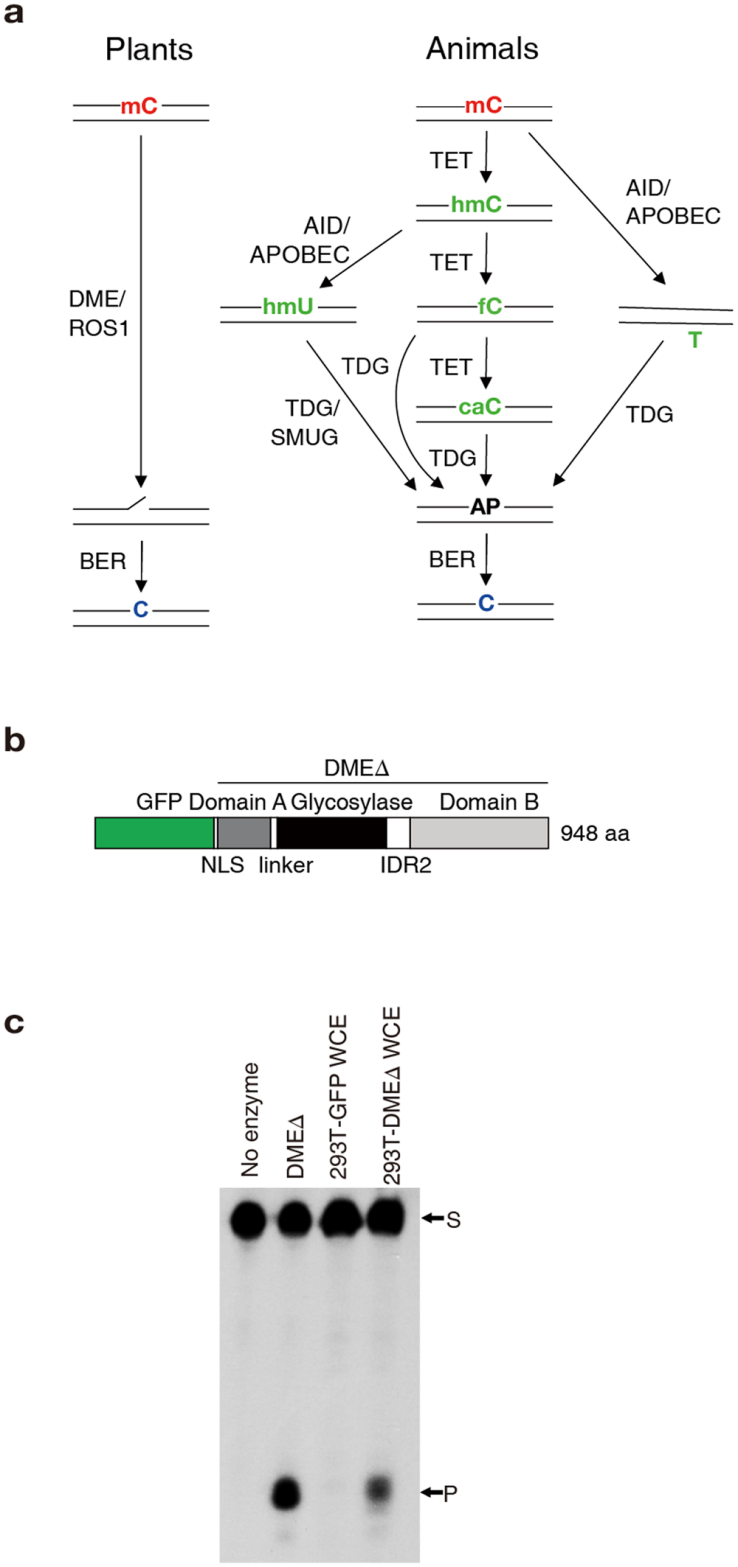
DMEΔ catalyses 5mC excision in HEK-293T cells. (**a**) Active DNA demethylation pathways in plants and animals. In plants, DME/ROS1 family DNA demethylase recognizes and excises 5mC from DNA forming a nick, which is then repaired through the BER and eventually replaced with unmethylated C. In animals, 5mC is successively converted to 5hmC, 5fC, and 5caC by TET enzymes prior to base excision. TDG is responsible for excision of 5fC or 5caC producing an abasic (AP) site, which is repaired and replaced with C through the BER. Alternative route may include conversions of 5mC and 5hmC into T and 5hmU, respectively, by AID/APOBEC deaminases prior to excision by TDG and SMUG. (**b**) Schematic representation of the GFP-DMEΔ fragment expressed in HEK-293T cells. IDR2, interdomain region 2 between the glycosylase domain and domains A and B. (**c**) 5mC excision activity in the whole cell extract (WCE) of the 293T-DMEΔ cells. A purified SUMO-DMEΔ protein was used as a positive control. The 35-mer oligonucleotide substrate containing 5mC at position 18 (S) and 5mC excision products (P) are indicated to the right of the panel. Blot image was cropped for better display.

DNA demethylation in animals requires successive base conversion of 5mC prior to its removal, whereas plants utilize 5mC DNA glycosylases (DNA demethylases) to directly remove it (Fig. 1a). In order to implement direct DNA demethylation activity in animal cells, we introduced *Arabidopsis* DME DNA demethylase into human embryonic kidney (HEK)-293T cells by transfection because of their reliable growth, transfection feasibility, and stable expression of exogenous genes. For expression of active DNA demethylase in HEK-293T cells, an engineered DMEΔN677ΔIDR1 fragment^19^, comprising only the domains essential for 5mC excision, was fused with a green fluorescent protein (GFP) and the cytomegalovirus nuclear localization sequence (NLS) (called GFP-DMEΔ hereafter) (Fig. 1b). The GFP-DMEΔ fusion protein was found to be localized in the nucleus (Supplementary Fig. 1), and the whole cell extract prepared from HEK-293T cells expressing GFP-DMEΔ (called 293T-DMEΔ hereafter) was able to catalyse the excision of 5mC from a double-stranded oligonucleotide substrate *in vitro*, whereas the extract obtained from the HEK-293T cells expressing only GFP (293T-GFP) did not process the methylated DNA substrate (Fig. 1c). This suggests that expression of *Arabidopsis* DME DNA demethylase in HEK-293T cells may confer catalytic activity of direct 5mC excision to cultured animal cells.

**Figure 2:**
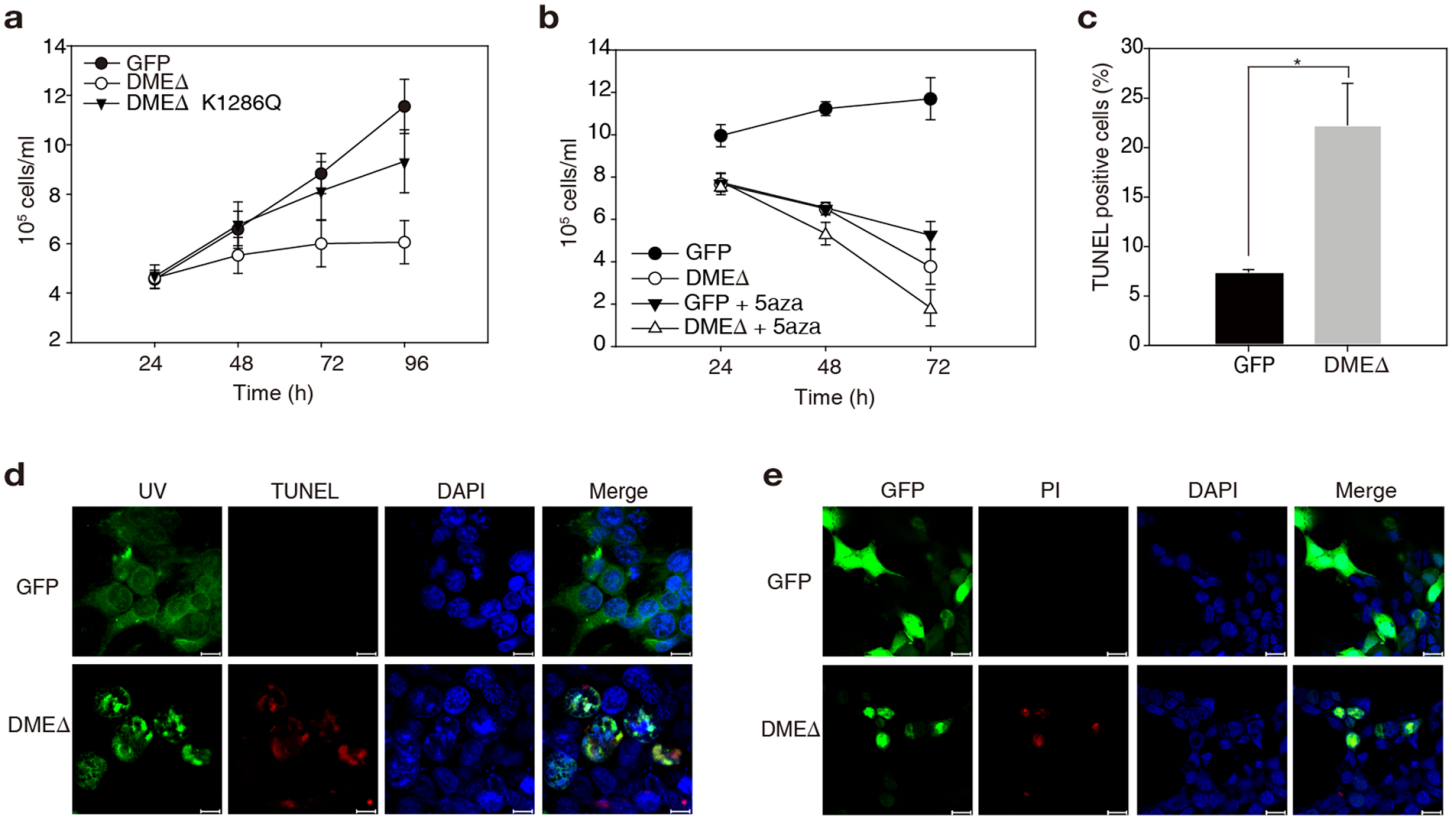
DMEΔ inhibits cell proliferation and causes DNA damage in HEK-293T cells. (**a**) Cell proliferation rates of the 293T-GFP, 293T-DMEΔ and 293T-DMEΔ(K1286Q) cells after transfection. DMEΔ(K1286Q) is a catalytically inactive fragment and was used as a negative control^19^. (**b**) Cell proliferation rates of the 293T-GFP and 293T-DMEΔ cells in the presence or absence of 25 μM 5aza in the culture medium. (**c**) Fractions of TUNEL-positive cells were analysed by flow cytometry. (**d**) TUNEL assays in the 293T-GFP and 293T-DMEΔ cells 48 h after transfection. Scale bar = 10 μm. (**e**) PI staining of the 293T-GFP and 293T-DMEΔ cells 48 h after transfection. Scale bar = 20 μm. Error bars represent mean ± S.D. of three independent experiments. *p < 0.05; paired sample *t*-test.

Notably, the proliferation rate of 293T-DMEΔ cells was significantly reduced compared to the HEK-293T cells expressing GFP only or catalytically inactive DMEΔ (K1286Q) (Fig. 2a). A similar reduction was observed when HEK-293T cells were treated with the DNMT inhibitor 5-azacytidine (5aza) (Fig. 2b). These observations suggest that abrupt DNA demethylation events, regardless of active or passive modes, have a negative effect on cell proliferation. Moreover, the 5aza-treated 293T-DMEΔ cells exhibited a more severe reduction in cell proliferation rate, indicating that DME expression and 5aza treatment have synergistic effects on cell proliferation inhibition (Fig. 2b).

### Direct 5mC excision induces DNA damage and cell death in mammalian cells

As a bifunctional DNA glycosylase, DME catalyses both 5mC excision and a strand cleavage, producing 3′-phosphor-α, β-unsaturated aldehyde (3′-PUA) and 3′-phosphate by successive β- and δ-elimination reactions, respectively^20–22^. Such lesions are intrinsically accompanied with DNA strand breaks acting as primary replication blocks^23^. Terminal deoxynucleotidyl transferase dUTP nick end labeling (TUNEL) assays detected significant DNA damage signals in 293T-DMEΔ cells (Fig. 2,c and d). DNA damage is one of the primary causes of apoptotic response^24^, but 293T-DMEΔ cells did not display typical characteristics of apoptosis such as cell shrinkage, nuclear condensation, DNA fragmentation and the cleavage of poly [ADP-ribose] polymerase 1 (PARP1) protein (Supplementary Fig. 2). Rather, propidium iodine (PI) staining showed that 293T-DMEΔ cells had increased membrane permeability (Fig. 2e), which is generally observed in cells that undergo necrosis^25^. These findings suggest that plant DNA demethylase induces DNA damage in mammalian cells leading to non-programmed cell death rather than triggering apoptotic response.

### Stress response genes are upregulated by DNA demethylase

**Figure 3:**
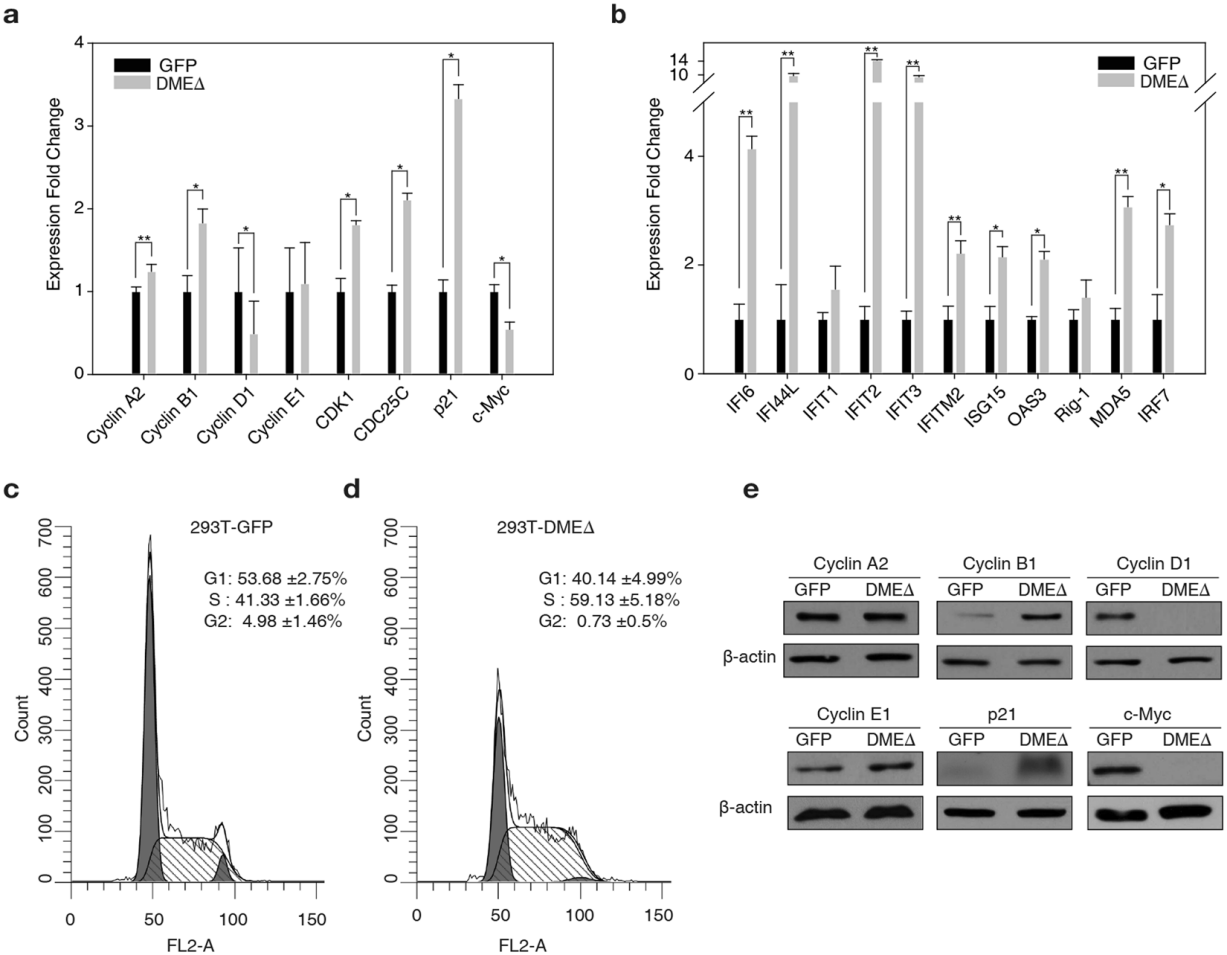
S phase arrest and dysregulation of the cell cycle components in the 293T-DMEΔ cells. (**a**), (**b**) qRT-PCR analysis of the cell cycle components (**a**) and ISGs (**b**) in the 293T-GFP and 293T-DMEΔ cells 48 h after transfection. (**c**), (d) DNA content was analysed by flow cytometry (FL2-A) in 293T-GFP (**c**) and 293T-DMEΔ cells (**d**) harvested 48 h after transfection. The proportion of DNA at S phase was calculated using the ModFit LT version 3.0 software. (**e**) Western blot analysis of cyclins A2, B1, D1 and E1, p21 and c-Myc in the 293T-GFP and 293T-DMEΔ cells. β-Actin was analysed as a loading control. Blot images were cropped for better display. Error bars represent mean ± S.D. of three independent experiments. *p < 0.05, **p < 0.005; paired sample *t*-test.

Aberrant DNA methylation is often associated with changes in gene expression. To understand the relationship between DME-induced active DNA demethylation and transcription, we obtained GFP-positive 293T-GFP and 293T-DMEΔ cells by fluorescence activated cell sorting (FACS) 48 h after transfection and performed gene expression analysis on a microarray platform (Supplementary Dataset 1). Compared to the 293T-GFP control, a total of 155 genes were upregulated and 42 genes downregulated in the 293T-DMEΔ cells (≥2-fold, p ≤ 0.05; Supplementary Fig. 3). The upregulated genes include a group of heat shock proteins (Hsps), cell cycle regulators, and unexpectedly, several interferon (IFN) response factors (Table 1). We verified their upregulation in the 293T-DMEΔ cells by quantitative real-time PCR (Fig. 3a,b, and Supplementary Fig. 4).

**Table 1:** List of upregulated and downregulated genes in 293T-DMEΔ cells.

Protein group and accession no.	Gene	Symbol	Locus	Fold change	p-value
Cell cycle components					
NM_000389	cyclin-dependent kinase inhibitor 1 A (p21, Cip1)	CDKN1A	chr6p21.2	2.82	0.031
NM_001135733	tumor protein p53 inducible nuclear protein 1	TP53INP1	chr8q22	2.75	0.002
NM_152562	cell division cycle associated 2	CDCA2	chr8p21.2	2.00	0.001
NM_031299	cell division cycle associated 3	CDCA3	chr12p13	1.96	0.0004
NM_001790	cell division cycle 25 homolog C (S. pombe)	CDC25C	chr5q31	1.90	0.001
NM_018101	cell division cycle associated 8	CDCA8	chr1p34.3	1.82	0.00006
NM_001170406	Cyclin-dependent kinase 1	CDK1	chr10q21.1	1.58	0.0005
NM_001130851	cyclin-dependent kinase inhibitor 3	CDKN3	chr14q22	1.56	0.001
NM_031966	cyclin B1	CCNB1	chr5q12	1.50	0.001
NM_002467	v-myc myelocytomatosis viral oncogene homolog (avian)	MYC	chr8q24.21	−1.60	0.001
NM_053056	cyclin D1	CCND1	chr11q13	−1.84	0.001
Interferon genes					
NM_006820	interferon-induced protein 44-like	IFI44L	chr1p31.1	14.62	0.0005
NM_001548	interferon-induced protein with tetratricopeptide repeats 1	IFIT1	chr10q23.31	5.93	0.0001
NM_001031683	interferon-induced protein with tetratricopeptide repeats 3	IFIT3	chr10q24	5.15	0.0002
NM_001547	interferon-induced protein with tetratricopeptide repeats 2	IFIT2	chr10q23.31	4.50	0.003
NM_006074	tripartite motif-containing 22	TRIM22	chr11p15	3.97	0.002
NM_022873	interferon, alpha-inducible protein 6	IFI6	chr1p35	3.88	0.0004
NM_001572	interferon regulatory factor 7	IRF7	chr11p15.5	2.95	0.001
NM_005101	ISG15 ubiquitin-like modifier	ISG15	chr1p36.33	2.44	0.0004
NM_014314	DEAD (Asp-Glu-Ala-Asp) box polypeptide 58	DDX58	chr9p12	2.35	0.0004
NM_006187	2′–5′-oligoadenylate synthetase 3, 100 kDa	OAS3	chr12q24.2	2.29	0.002
NM_006435	interferon induced transmembrane protein 2 (1-8D)	IFITM2	chr11p15.5	2.10	0.001
Heat shock proteins					
NM_002155	heat shock 70 kDa protein 6 (HSP70B’)	HSPA6	chr1q23	23.30	0.00006
NM_005345	heat shock 70 kDa protein 1 A	HSPA1A	chr6p21.3	8.92	0.0001
NM_006145	DnaJ (Hsp40) homolog, subfamily B, member 1	DNAJB1	chr19p13.2	5.63	0.00009
NM_021979	heat shock 70 kDa protein 2	HSPA2	chr14q24.1	3.71	0.0007
NM_001540	heat shock 27 kDa protein 1	HSPB1	chr7q11.23	3.50	0.0001
NM_007034	DnaJ (Hsp40) homolog, subfamily B, member 4	DNAJB4	chr1p31.1	1.94	0.0003
NM_006644	heat shock 105 kDa/110 kDa protein 1	HSPH1	chr13q12.3	1.91	0.00003
NM_002154	heat shock 70 kDa protein 4	HSPA4	chr5q31.1	−1.53	0.002

Considering the proposed role of DNA demethylase as a transcription activator, there are two possible explanations for transcription upregulation: 1) direct transcriptional activation as a result of DNA demethylation and 2) indirect upregulation via DNA demethylation-triggered signalling cascades. To distinguish between the two possible scenarios, we compared the genome-wide DNA methylation patterns of 293T-GFP and 293T-DMEΔ cells 48 h after transfection using a Human Methylation 450 K Bead Chip array (Supplementary Dataset 2). Genome-wide DNA methylation profiles between 293T-GFP and 293T-DMEΔ cells are highly similar to each other, indicating that DME expression did not induce substantial DNA demethylation in HEK-293T cells (Supplementary Fig. 5a and b). Moreover, there was little correlation between gene expression and DNA methylation levels. Locus-specific bisulfite sequencing also revealed no significant changes in DNA methylation levels of representative hypo-, intermediate-, and hypermethylated genes in the 293T-DMEΔ cells (IFI6, IFI44L, and MLH3, respectively; Supplementary Fig. 5c–e), even though they were all transcriptionally upregulated by DME expression. This indicates that transcriptional activation by DME DNA demethylase is a late, indirect response rather than a direct consequence of the removal of DNA methylation at the target loci.

### Upregulation of heat shock proteins by DME expression

Many Hsps are upregulated in response to a variety of stress stimuli including DNA damage^26, 27^, and we observed a substantial increase in the expression levels of diverse Hsp genes in the 293T-DMEΔ cells (Table 1 and Supplementary Fig. 4b). In particular, genes encoding Hsp70s (HSPA6, HSPA1A, and HSPA2), Hsp40 (DNAJB1) and Hsp27 (HSPB1) were highly expressed (>3.5 fold). Although the detailed mechanisms by which Hsps mediate DNA damage signals are still elusive, these findings suggest that harmful DNA lesions associated with DME-induced active DNA demethylation may trigger heat shock response as well as the inhibition of apoptosis partly because of their antiapoptotic function^28^.

### Direct 5mC excision by DNA demethylase induces cell cycle arrest at S phase

One of the key mechanisms that control cell cycle progression is the regulation of the activity of essential cell cycle proteins such as cyclins and cyclin-dependent kinases (CDKs) and their abundance^29^. The S phase, during which DNA synthesis occurs, is highly sensitive to genotoxic DNA damage. A significant portion of 293T-DMEΔ cells exhibited S phase arrest 48 h after transfection (Fig. 3c and d; 59.13% vs. 41.33% in the control). It is plausible that DME DNA demethylase generates damage signals that lead to the checkpoint activation and the inhibition of cell cycle progression, and eventually restrict cell proliferation with S phase arrest (Fig. 2a). This can be partly explained by the production of excessive abasic sites and SSBs, as both serve as primary replication blocks preventing DNA synthesis^23^. Alternatively, DME expression may induce a change in the abundance of essential cell cycle regulators or factors that are key to cycle management^30^. A decrease in the transcription level of cyclin D1 but an increase of cyclin B1 was observed in 293T-DMEΔ cells 48 h after transfection, whereas cyclin A2 and cyclin E were expressed at almost the same level in both 293T-GFP and 293T-DMEΔ cells (Fig. 3a). The level of CDK1 expression was slightly higher in 293T-DMEΔ cells. Remarkably, at the protein level, a substantially lower amount of cyclin D1 and more cyclin B1 were present in the 293T-DMEΔ cells compared to the 293T-GFP control (Fig. 3e). These observations indicate the dysregulation of the major cell cycle proteins in the cells that express DME DNA demethylase. The 293T-DMEΔ cells also retained a high level of p21, a CDK inhibitor that restrains cyclin-CDK complexes and negatively regulates cell cycle progression while promoting G_1_/S arrest^29^. By contrast, c-Myc negatively regulating p21 transcription^31^ was significantly depleted (Fig. 3e). This suggests that abnormal p21 activation and c-Myc downregulation may also cause S arrest and growth retardation observed in 293T-DMEΔ cells.

### Interferon response triggered by DME expression

**Figure 4:**
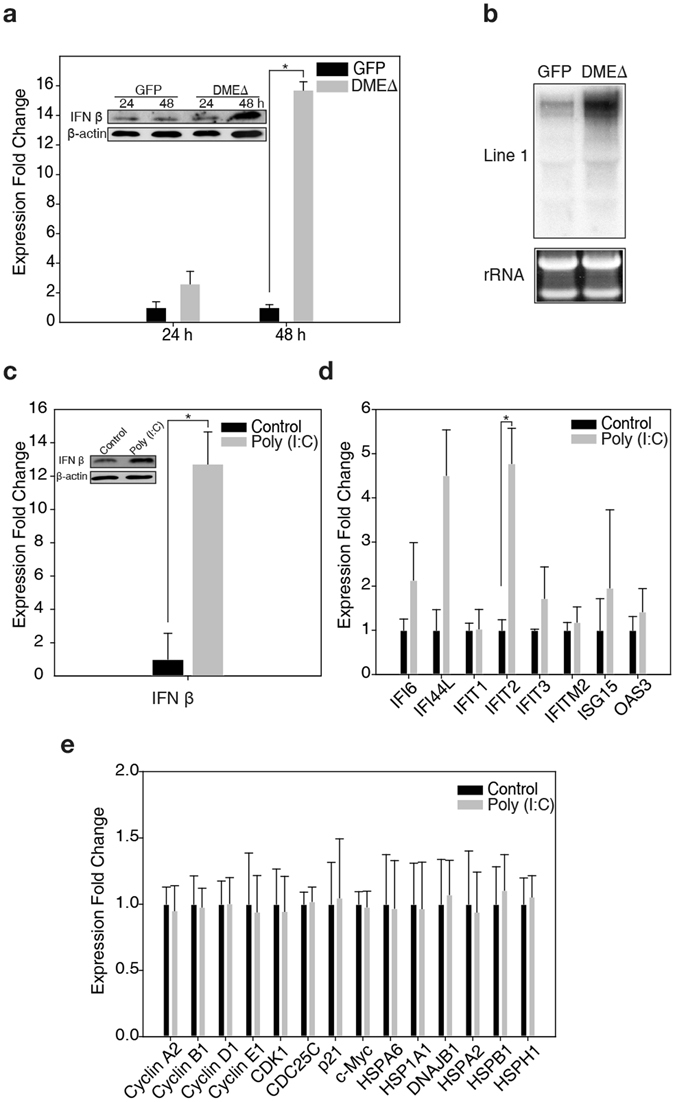
IFN signalling and the antiviral response result from the DME-induced dsRNA formation. (**a**) Abundance and expression of IFN β in 293T-GFP and 293T-DMEΔ cells. (**b**) Northern blot analysis for the L1 elements in 293T-GFP and 293T-DMEΔ cells. (**c**) Abundance and expression of IFN β in the HEK-293T cells 36 h after poly (I:C) treatment. (**d**), (**e**) qRT-PCR analysis of the ISGs (**d**), and cell cycle components and HSPs (**e**) in the HEK-293T cells 36 h after the poly (I:C) treatment. All blot and gel images were cropped for better display. Error bars represent mean ± S.D. of three independent experiments. *p < 0.05; paired sample *t*-test.

Notable among the upregulated genes in the 293T-DMEΔ cells were IFN-stimulated genes (ISGs) such as IFNα-inducible protein 6 (IFI6), IFN-induced protein 44-like (IFI44L), IFI with tetratricopeptide repeats 1 (IFIT1), IFIT2, IFIT3, IFI transmembrane protein 2 (IFITM2), ISG15, 2′–5′-oligoadenylate synthase 3 (OAS3), and retinoic acid-inducible gene I (RIG-I) (Table 1 and Fig. 3b). IFNs are signalling proteins secreted from infected cells and induce cell-intrinsic antimicrobial states^32, 33^. The 293T-DMEΔ cells were found to produce a high level of IFN β 48 h after transfection (Fig. 4a). The canonical type I IFN signalling induces the expression of a number of ISGs responsible for establishing the cellular antiviral state^33^. Interestingly, many of the ISGs induced by DME are implicated in RNA processing. For example, IFITs, which are highly upregulated upon viral infection^34^, were significantly upregulated in 293T-DMEΔ cells (Table 1 and Fig. 3b). Other important players include DEAD-box RNA helicase proteins such as retinoic acid-inducible gene I (RIG-I) and melanoma differentiation-associated gene 5 (MDA5), which are known to recognize viral RNAs to initiate the antiviral state^35^. OAS3 is responsible for degrading viral RNAs by activating RNase L^36^.

### Activation of dsRNA sensor to trigger antiviral response

The recognition of viral RNAs by RIG-I and MDA5 initiates signalling cascades for innate immune response, and IFITs directly bind to viral RNA for translation inhibition^37^. Thus, it is reasonable to speculate that some viral RNA-like patterns are generated during DME-induced active DNA demethylation and subsequently initiate the defence program, even in the absence of viral infection. It is worth noting that some retrotransposable elements such as long interspersed nuclear element 1 (L1) are highly abundant in the human genome (~17% with >500,000 copies) and their promoter regions are heavily methylated and silenced^38^. We found that significant amounts of L1 transcripts were detected in 293T-DMEΔ cells (Fig. 4b). It was also shown that DME induced the upregulation of a subclass of human retrotransposon endogenous retroviral element (ERV)-K, one of the most characterized ERVs that are still active in the human genome and responsible for cancer development^39^ (Supplementary Fig. 6a,b). Tag-assisted sense/antisense transcript detection (TASA-TD) PCR^40^ verified that both L1 and ERV-K were bidirectionally transcribed in 293T-DMEΔ cells (Supplementary Fig. 6c). Thus, it is plausible that DME-induced upregulation of retrotransposons such as L1 and ERVs generates both sense and antisense transcripts from different loci, subsequently forming dsRNAs between the complementary transcripts to activate the RIG-I/MDA5 cytosolic RNA sensors for the activation of IFN signalling. It was also shown that the HEK-293T cells treated with dsRNA analogue polyinosinic:polycytidylic acid (poly (I:C)) produced IFN β (Fig. 4c) and activated a subset of ISGs (Fig. 4d), whereas expressions of the cell cycle components and Hsps were not significantly affected (Fig. 4e). These findings indicate that the activation of antiviral response involving dsRNA is part of the cellular response triggered by DME but can be uncoupled from other responses such as cell cycle dysregulation and heat shock response. Moreover, the activation of L1 and ERV-K is quite similar to the consequence of passive DNA demethylation achieved by DNMT inhibition, where the dsRNAs from a panel of ERVs are proposed to be the most likely trigger for IFN signalling and antiviral response^41, 42^. Our data suggest that fundamental mechanistic differences exist between the cellular responses to DME DNA demethylase expression and DNMT inhibition, although common signal cascades operate in response to loss of global DNA methylation.

## Discussion

Here we showed that the introduction of *Arabidopsis* DME DNA demethylase into mammalian cells allowed the acquisition of unprecedented direct 5mC excision activity, thereby bypassing TET-mediated oxidatfion of 5mC into other bases for DNA demethylation. Expression of DME inevitably induces DNA damage, due to the bifunctional DNA glycosylase activity of this family of enzymes that leads to the production of numerous abasic sites and DNA strand breaks during 5mC excision^4, 19, 20^. These DNA lesions are likely to act as DNA damage signals in mammalian cells causing the inhibition of cell proliferation and S arrest, accompanied with changes in the abundance of many essential cell cycle regulators. Even though the detailed mechanisms of how DME induces cell cycle arrest and inhibits cell proliferation should be further investigated, this study suggests that the implementation of DNA demethylase can be clinically useful to suppress cell division activity of many cancer cells.

5Aza is known to induce ATM/ATR-mediated DNA double-strand break response and apoptosis^43^. Indeed, diverse genotoxic DNA damages such as DSBs, base adducts, crosslinks and replication blocks activate the DNA damage response in mammalian cells. However, we did not observe in DME expressing cells the activation of ATM, ATR or Chk1 and Chk2 effector kinases in the ATM/ATR DNA damage checkpoint pathway (Supplementary Dataset 1). Therefore, it is thought that DME produces unique DNA damage lesions and induces S phase arrest independently of ATM/ATR-dependent pathway. Although 5mC usually occurs symmetrically at CpG dinucleotides, DME DSB production is unlikely to occur since DME has poor activity for excision of both 5mCs at symmetrically methylated CpG sites^4^. Therefore, the main DNA lesions responsible for DNA damage response in DME-expressing cells are likely to be SSBs associated with 3′-PUA and 3′-phosphate arising from 5mC excision, which are supposedly processed by canonical BER enzymes such as APE1 in mammalian cells, but their copious production may exceed the repair capacity and ultimately act as DNA damage signals. SSBs are sometimes detected by PARP1 binding and activation, which promotes rapid activation of downstream repair factors^23^. However, we did not observe PARP1 cleavage, which is one of the molecular signatures in apoptotic cells, proposing that functional PARP1 may be responsible for the recognition of SSBs generated by DME. Upregulation of HSPs involved in DNA damage repair may also facilitate the repair of DME-induced SSBs, even though we cannot rule out the possibility that exogenous protein expression requires HSPs to assist proper protein folding.

Although DME isolated from HEK-293T cells was able to excise 5mC from DNA *in vitro* (Fig. 1c), we did not observe significant hypomethylation at any specific loci in 293T-DMEΔ cells (Supplementary Fig. 5a and b). We presume the reason is that DME is likely to remove 5mC randomly throughout the genome rather than to target specific sites. DME has little sequence preference for 5mC excision, and 5mC is found in approximately 1.5% of human genomic DNA^44^. Therefore, a chance to detect discernible DNA demethylation at individual sites will be extremely low without triggering severe DNA damage, particularly considering the cytotoxicity of DME expression accompanied with the accumulation of harmful 5mC excision intermediates^20^.

As there are numerous 5mC residues in the mammalian cells as potential targets of DME, we hypothesized that global DNA methylation changes occurred by DME expression in a stochastic manner, randomly activating a wide panel of genes with diverse cellular functions. In fact, our microarray and DNA methylation array data showed that there is little correlation between DNA demethylation and transcription activation, suggesting that many genes are upregulated indirectly rather than as a direct consequence of DNA demethylation *per se*. Interestingly, retrotransposable elements such as L1 and ERV-K were upregulated by DME, potentially serving as viral mimics to induce a cellular antiviral response. Recent studies also showed that 5aza-mediated passive DNA demethylation upregulates diverse ERV retrotransposons^41, 42^. It is remarkable that although active and passive DNA demethylation mechanisms are fundamentally different, DME-induced active DNA demethylation and 5aza-mediated passive DNA demethylation bring about similar outcomes such as upregulation of retrotransposons and the activation of IFN signalling mediated by cytoplasmic dsRNA sensing, leading to the onset of antiviral state.

Notably, the IFN signaling pathway is thought to play a central role in counteracting such a pseudo-antiviral condition by upregulating a number of ISGs required for viral defence. It was shown that many ISGs responsive to type I IFN are upregulated as late-response genes 5 days after 5aza treatment with a peak at 24 days after treatment in colorectal cancer cells^42^. In addition, many ISGs are upregulated at least 7 days after 5aza treatment in ovarian cancer cells, in which IFNβ is crucial for 5aza-induced ISG upregulation^41^. However, unlike 5aza-treated passive DNA demethylation, DME-induced active DNA demethylation required only 2 days for the upregulation of ISGs, for which IFNβ also appeared to play an important function. These and our findings suggest that both 5aza treatment and DME expression elicit the common IFN signalling cascade but the latter has more immediate effects on the onset of antiviral state because DME-mediated active DNA demethylation does not require cell division to achieve loss of DNA methylation sufficient to trigger the response. All these studies also demonstrate that one of the major functions of DNA methylation is to repress the expression of potentially harmful repeat elements that would otherwise severely compromise the genome integrity when expressed.

Recent studies report that a human TET1 fused with the genome editing modules such as a Transcription Activator-Like Effector (TALE) and a Clustered Regularly Interspaced Short Palindromic Repeat (CRISPR)/dead CRISPR-associated protein 9 (dCas9) system can activate endogenous target genes^45–47^. Therefore, proteins that modify 5mC in the genome, including DME, can be utilized for epigenome manipulation by adopting the current genome editing technologies^48^. In addition, as DME also has excision activity towards 5hmC^49^, it would be helpful to use DME to discern the role and effect of 5hmC on the DNA methylation dynamics in vertebrates, which is currently largely unknown.

## Methods

### Cloning

A cytomegalovirus NLS fragment was prepared by annealing the complementary DG104 and DG105 oligonucleotides and inserting them into the pEGFP-C1 vector at Bgl II and Sal I sites (Clontech). A DMEΔN677ΔIDR1::lnk fragment^19^ (called DMEΔ hereafter) was amplified by PCR using the DG49 and DG65 primers, and then cloned into the Sal I and Bam HI sites of the pEGFP-C1 vector with the NLS to produce pEGFP-NLS-DMEΔ. The EGFP-NLS-DMEΔ fragment was PCR-amplified with the DG392 and DG394 primers, digested with Nhe I and Pme I, and then cloned into the corresponding sites of the pCDNA3.1/hygro/lacZ vector to generate pCDAN3.1-GFP-NLS-DMEΔ. As a control, the pCDNA3.1-GFP construct was produced using essentially the same procedure except that the GFP fragment was amplified using the DG392 and DG393 primers. Primer sequences are provided in Supplementary Table 1.

### Cell culture and cell proliferation assay

HEK-293T cells were grown in Dulbecco’s modified Eagle’s medium (DMEM) (Hyclone) supplemented with 10% fetal bovine serum (Hyclone) and a 1% antibiotic/antimycotic solution (Hyclone). For the cell proliferation assay, HEK-293T cells (2 × 10^5^ cell/plate) were seeded on a 60 mm cell culture plate, grown for 24 h, and then transfected with the pCDNA3.1-GFP, -DMEΔ, and -DMEΔ(K1286Q) plasmids using the Lipofectamine 2000 Reagent (Invitrogen) according to the manufacturer’s protocol. The DMEM medium containing 200 μg/mL of hygromycin B (AG Scientific) was replaced every 24 h. The number of cells was counted every 24 h after transfection using a hemocytometer for up to four days. The HEK-293T cells harbouring the pCDNA3.1-GFP and -DMEΔ plasmids are named 293T-GFP and 293T-DMEΔ, respectively. Another set of cell culture plates (5 × 10^5^ cell/plate) was prepared in parallel and treated with 25 μM 5-azacytidine (5aza) (MP Biomedicals) 24 h after transfection. The DMEM medium containing 200 μg/mL of hygromycin B (AG Scientific) (+25 μM 5aza, if necessary) was freshly changed every 24 h. The number of cells was counted every 24 h after 5aza treatment for up to three days.

### Fluorescence activated cell sorting (FACS) analysis

The 293T-GFP and 293T-DMEΔ cells were prepared as described in the cell culture section and harvested 48 h after transfection. The DMEM medium containing 200 μg/mL of hygromycin B (AG Scientific) was replaced 24 h after transfection. The GFP-positive cells were collected using a FACS Aria III (BD Biosciences). For the cell cycle analysis, the 293T-GFP and 293T-DMEΔ cells were harvested by centrifugation 48 h after transfection. The cells were resuspended in 1 mL of ice-cold 70% ethanol and mixed with gentle agitation overnight at 4 °C. The fixed cells were washed with phosphate-buffered saline (PBS) and harvested by centrifugation at 300 g for 6 min. For the propidium iodide (PI) staining, the pellets were resuspended in 0.5 mL of binding buffer (10 mM HEPES, pH 7.4, 140 mM NaCl, and 2.5 mM CaCl_2_) and 10 µL of RNase A (80 µg/mL, Sigma), and then stained with 25 µL of PI solution (50 μg/mL, Sigma) by incubating them on a rocker for 30 min in the dark at room temperature. The PI-stained cells were subjected to analysis on a FACS Calibur (BD Biosciences) flow cytometer and all flow cytometry histograms were analysed by the ModFit LT version 3.0 software (Verity Software).

### Microscopy analysis

To examine the membrane integrity with PI staining, the 293T-GFP and 293T-DMEΔ cells were stained with 8 μg/mL PI (Sigma) solution for 10 min in the dark at room temperature. The cells were rinsed twice with 3% bovine serum albumin (BSA) in PBS and stained with 300 nM 4′,6-diamidino-2-phenylindole (DAPI, Invitrogen) in 1 mL of PBS for 5 min at room temperature. The cells were washed twice with 3% BSA in PBS and mounted in fluorescent mounting medium (Dako). The cells were examined under a LSM700 confocal laser scanning microscope (Carl Zeiss), and the images processed and analysed with the ZEN 2009 Software (Carl Zeiss).

### TUNEL assay

HEK-293T cells (1 × 10^5^ cells/slip) were place onto a poly-L-lysine (Sigma) coated cover glass in 3 mL of DMEM medium, incubated for 24 h, and transfected with the pCDNA3.1-GFP and -DMEΔ plasmids as described in the cell culture section. Forty-eight hours after transfection, the cells were fixed in 100% methanol for 15 min at −20 °C. The fixed cells were washed with 3% BSA in PBS and incubated with 45 μL of freshly prepared terminal deoxynucleotidyl transferase (TdT) dUTP nick end labelling (TUNEL) reaction buffer (Roche) (1x TdT reaction buffer and 5 mM CoCl_2_) for 10 min at 37 °C in humidity chamber. The cells were then subjected to the TUNEL reaction with 400 U of TdT enzyme (Roche) and 20 μM BrdUTP (Invitrogen) in 50 μL of TUNEL reaction buffer for 1 h at 37 °C in humidity chamber. After three 5 min washes with 3% BSA in PBS, the cells were incubated with an AlexaFluor 594-conjugated BrdUTP antibody (Life Technologies, B35132) in dilution buffer (5% bovine serum albumin and 0.3% Triton X-100 in PBS) for 1 h in the dark at room temperature. Finally, the cells were washed with 3% BSA in PBS three times, stained with DAPI, and mounted in fluorescent mounting medium (Dako). The cells were examined under the LSM700 confocal laser scanning microscope (Carl Zeiss), and the images processed and analysed with the ZEN 2009 Software (Carl Zeiss). Fractions of TUNEL-positive cells were analysed by FACS following the procedure described above with the aid of the Flowjo software (Tree Star) for calculation.

### polyinosinic:polycytidylic acid (poly (I:C)) transfection

HEK-293T cells (5 × 10^5^ cell/plate) were seeded on a 60 mm cell culture plate, grown for 24 h, and then transfected with poly (I:C) (10 μg/mL, Sigma). Cells were harvested 36 h after transfection and subjected to downstream analysis.

### *in vitro* 5mC glycosylase assay

The *in vitro* reaction essentially followed the procedure by Mok *et al*.^19^ with slight modifications. Two days after transfection, GFP-positive cells were FACS-sorted, harvested by centrifugation at 300 g for 5 min at 4 °C, and then resuspended in 100 µL of extraction buffer (50 mM Tis-HCl, pH 7.4, 100 mM NaCl, 10% glycerol, 0.1 mM dithiothreitol, a complete protease inhibitor cocktail tablet, Roche). Cells were lysed by sonication for 2 min on ice (output power, 4; duty cycle, 50%; Branson Sonifer 250). The lysate was centrifugated at 15,000 rpm for 20 min at 4 °C, and the whole cell extract (10 μg) was incubated with radio-labelled oligonucleotide substrate^19^ (13.3 nM) in 15 μL reaction with 10 mM Tris-HCl, pH 7.4, 50 mM NaCl, 1 mM dithiothreitol, 0.1 mM EDTA, pH 8.0 at 37 °C for 4 h. A purified SUMO-DMEΔN677ΔIDR1::lnk protein (50 ng) was used as a positive control.

### Western blot analysis

The 293T-GFP and 293T-DMEΔ cells were prepared as described in the FACS analysis section. The cells were lysed in 10 mL of lysis buffer (50 mM Tris-HCl, pH 8.0, 150 mM NaCl, 0.5% Nonidet P-40, and a complete protease inhibitor cocktail tablet, Roche) on ice for 30 min. The cell lysates were collected and separated on a 10% SDS-polyacrylamide gel. The gel was blotted onto a Hybond C membrane (GE Healthcare). The membrane was blocked with TBST (20 mM Tris-HCl, pH 7.4, 137 mM NaCl, and 0.1% Tween-20) containing 3% BSA for 1 h at room temperature and then incubated with the primary antibody (described below) solution overnight at 4 °C. After washing with TBST, the membrane was incubated with a horseradish peroxidase (HRP)-conjugated secondary antibody for 1 h at room temperature. The signals were detected using the SuperSignal West Pico Chemiluminescent Substrate (Thermo Scientific) and visualized by exposure onto an X-ray film (Fujifilm). The primary antibodies used for western analysis were against cyclin D1 (1:1,000, Santa Cruz Biotechnology, SC-20044), cyclin A (1:5,000, Santa Cruz Biotechnology, SC-596), cyclin B1 (1:1,000, Santa Cruz Biotechnology, SC-245), p21 (1:500, Santa Cruz Biotechnology, SC-6246), c-Myc (1:500, Santa Cruz Biotechnology, SC-746), IFN β (1:500, Abcam, ab6979), PARP (1:1,000, Cell Signaling Technology, 9542), and β-actin (1:10,000, Santa Cruz Biotechnology, SC-47778). Goat anti-mouse IgG-HRP (1:10,000, Santa Cruz Biotechnology, SC-2005) and goat anti-rabbit IgG-HRP (1:10,000, Santa Cruz Biotechnology, SC-2004) were correspondingly used as secondary antibodies.

### Microarray analysis

The microarray analysis was performed on a Human Genome U133 Plus 2.0 Array Platform (Affymetrix). The 293T-GFP and 293T-DMEΔ cells were prepared as described in the FACS analysis section. The GFP-positive cells were collected using a FACS Aria III (BD Science), and the total RNA was isolated using an RNeasy Mini Kit (Qiagen). Labelling and hybridization to the microarray were performed according to the manufacturer’s instructions. The arrays were scanned with an Affymetrix GeneChip Scanner 3000 7 G. The data were extracted with Affymetrix Command Console 1.1, and normalized using the MAS5 algorithm. Finally, the gene expression matrix was calculated using the R Affy package (v.2.11.1).

### Quantitative real time PCR analysis

The cells were harvested and the total RNA was extracted using an RNeasy Mini Kit (Qiagen). First-strand cDNA synthesis was performed using 1 μg of total RNA with Oligo(dT) primers (Invitrogen) and SuperScript III Reverse Transcriptase (Invitrogen) according to the manufacturer’s instructions. The products were subsequently diluted to a final concentration of 30 μg/mL in nuclease-free water. The qRT-PCR reaction was performed in a 10 μL volume with the Rotor-Gene SYBR Green PCR Kit (Qiagen) containing 60 ng of template cDNA and 200 nM each of gene-specific primers. The reaction was performed at 95 °C for 10 min followed by 40–50 cycles of 94 °C for 20 s, 60 °C for 20 s, and 72 °C for 30 s on a Rotor-Gene Q cycler (Qiagen). The data analysis was performed as previously described^50^. The sequences of the primers used in the qRT-PCR analysis are provided in Supplementary Table 2.

### Northern blot analysis

Total RNA was extracted from the 293T-GFP and 293T-DMEΔ cells 48 h after transfection and transferred onto a Hybond N^+^ membrane (GE Healthcare). The L1 element (accession no. M19503) was amplified by PCR using the DG2106 and DG2107 primers, and randomly labelled with [α-^32^P]dCTP (Perkin Elmer) using a Klenow fragment (3′ → 5′ exo^−^) (New England Biolabs). After hybridization, the membrane was exposed to a phosphorimaging screen (Fujifilm) and the radioactivity was measured with a BAS-5000 Phosphorimager (Fujifilm). Primer sequences are provided in Supplementary Table 1.

### Methylation analysis

GFP-positive 293T-GFP and 293T-DMEΔ cells were isolated as described in the FACS analysis section. Genome-wide DNA methylation analysis was performed on a Human Methylation 450 K BeadChip platform (Illumina)^51^. Briefly, 500 ng of each gDNA sample was bisulfite-converted with the EZ DNA Methylation Kit (Zymo Research). 200 ng of bisulfite-converted DNA was amplified and hybridized onto Infinium Human Methylation 450 K BeadChip (Illumina) according to the manufacturer’s protocol, and scanned using an Illumina iScan scanner (Illumina). Raw fluorescence intensity values were normalised using the Illumina GenomeStudio (V2011) Methylation Module. Normalised intensities were used to calculate β-values. The β-value represents the percentage of methylated cytosines at the locus, ranging from 0 (no methylation) to 1 (complete methylation). The BeadChip methylation data are provided as a Supplementary Dataset 2 and deposited in the GEO database with the Accession Number GSE96864. For locus-specific analysis, bisulfite conversion was conducted using the EpiTect Fast Bisulfite Conversion Kit (Qiagen). The bisulfite-treated fragments were amplified by PCR using the primers specific for IFI6, IFI44L and MLH3 genes. The PCR products were cloned into the RBC T-A Cloning Vector (Real Biotech Corporation) and sequenced. The DNA methylation patterns were analysed using the CyMATE program (http://www.cymate.org). Primer sequences for bisulfite sequencing are provided in Supplementary Table 3.

### TAG-aided sense/antisense transcript detection (TASA-TD) PCR

The experiment was essentially performed as previously described^40^. Briefly, 48 h after transfection, total RNA was extracted from FACS-sorted GFP-positive cells using Trizol (Invitrogen) and the first strand cDNA was synthesized. Strand-specific PCR was performed for L1, ERV-K and β-actin genes using TAG and gene-specific sense and antisense primers. Quantitation of PCR products were performed using ImageJ (http://imagej.nih.gov). Primer sequences for TASA-TD PCR are provided in Supplementary Table 4.

### Data availability

Transcriptome and DNA methylome data can be accessed at GEO database (Accession Number GSE96863, GSE96864).

## Electronic supplementary material

Supplemental Figures

Dataset 1

Dataset 2
