# Supplementary material for: DEMETER plant DNA demethylase induces antiviral response by interferon signalling in animal cells
Source: Sci Rep. 2017 Aug 22;7:9160. doi: 10.1038/s41598-017-08827-9 (PMC5567224; doi:10.1038/s41598-017-08827-9)
Supplement: Supplementary file 1 — Supplemental Figures [file 41598_2017_8827_MOESM1_ESM.pdf]

DEMETER plant DNA demethylase induces antiviral response by interferon signalling in animal cells

Young Geun Mok[1], Ki Young Choi[2], Seung Hwan Hong[2], and Jin Hoe Huh[1]

[1]Interdisciplinary Program in Agricultural Genomics, Department of Plant Science, Plant Genomics and Breeding Institute, and Research Institute of Agriculture and Life Sciences, Seoul National University, Seoul 08826, Korea

[2]School of Biological Sciences, Seoul National University, Seoul 08826, Korea

Correspondence to Jin Hoe Huh (email: huhjh@snu.ac.kr)

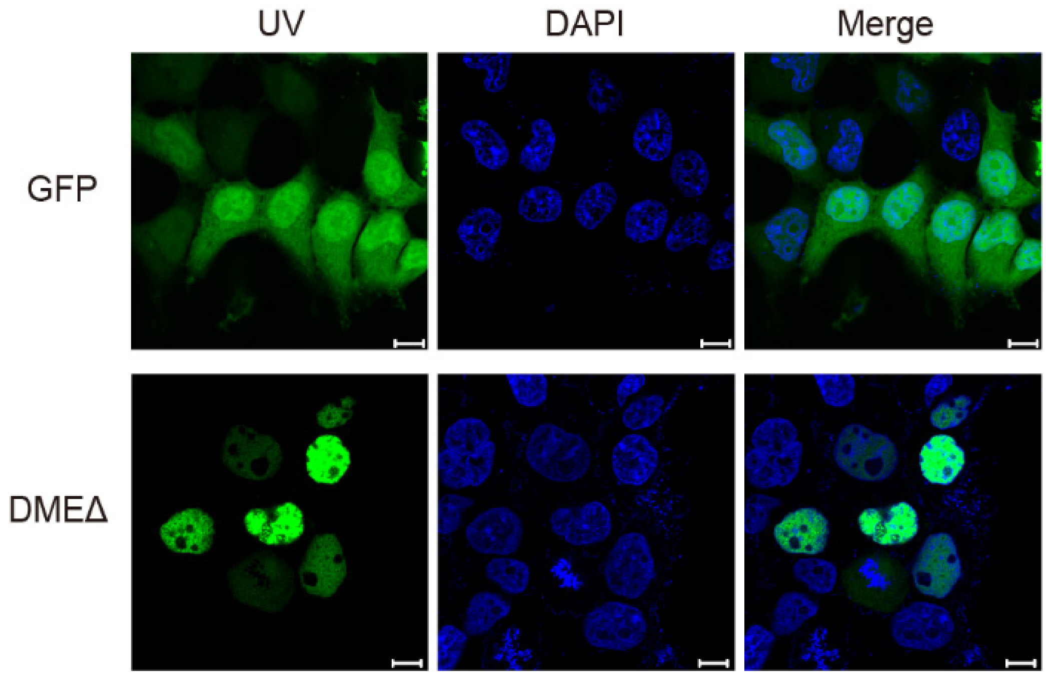

Supplementary Figure 1. Subcellular localizations of GFP and GFP-DMEΔ proteins in HEK-293T cells. GFP fluorescence signals (green) indicate subcellular localizations of GFP and GFP-DMEΔ proteins. DAPI (blue) signals indicate nuclei. Images were obtained 48 h after transfection. Scale bar = 10 μm.

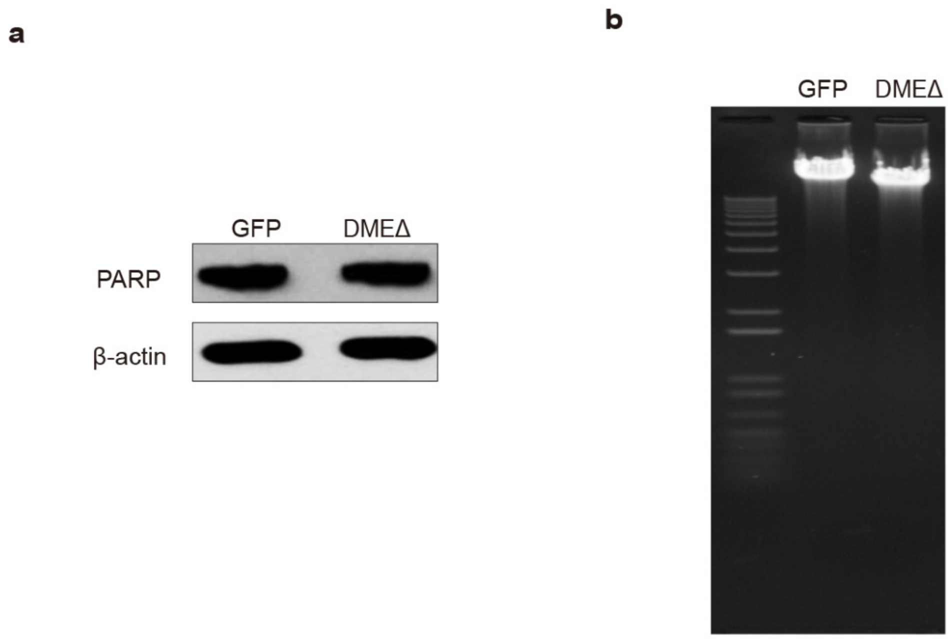

Supplementary Figure 2. Analysis of apoptotic molecular signatures in 293T-GFP and 293T-DMEΔ cells. (a) Western blot analysis of PARP proteins in 293T-GFP and 293T-DMEΔ cells. **(b)** Electrophoresis of genomic DNA obtained from 293T-GFP and 293T-DMEΔ cells on an agarose gel. All samples were prepared from 293T-GFP and 293T-DMEΔ cells 48 h after transfection.

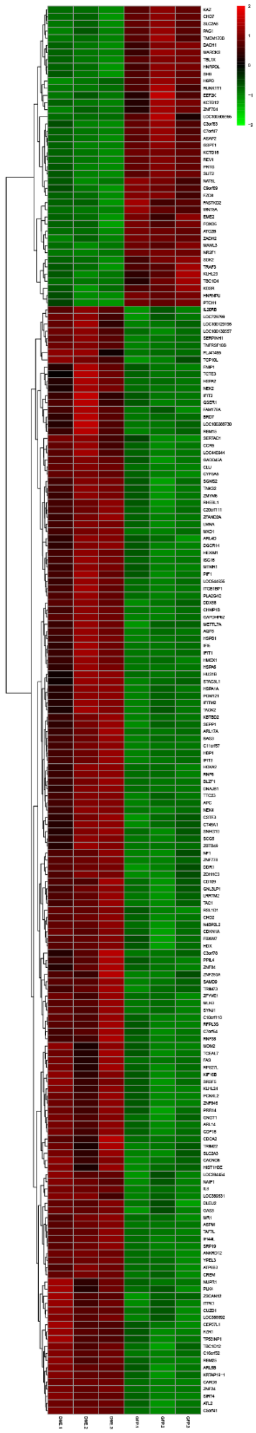

Supplementary Figure 3. Hierarchical clustering of differentially expressed genes in 293TGFP and 293T-DMEΔ cells. A total of 155 genes were upregulated and 42 down-regulated in 293T-DMEΔ cells compared to 293T-GFP cells (≥ 2-fold, p ≤ 0.05).

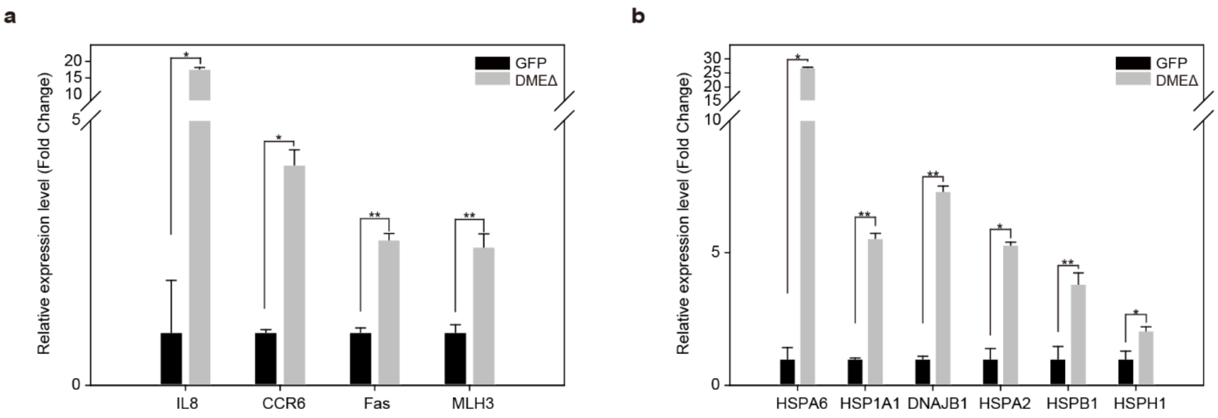

Supplementary Figure 4. Validation of gene expression levels by qRT-PCR. (a) Expression levels of the genes that are functionally unrelated but revealed to be upregulated in the 293TDMEΔ cells. **(b)** Expression levels of Hsp genes upregulated in the 293T-DMEΔ cells. Samples were collected and analyzed 48 h after transfection. Error bars represent mean ±S.D. of three independent experiments. *$p < 0.05$, **$p < 0.005$; paired sample t-test.

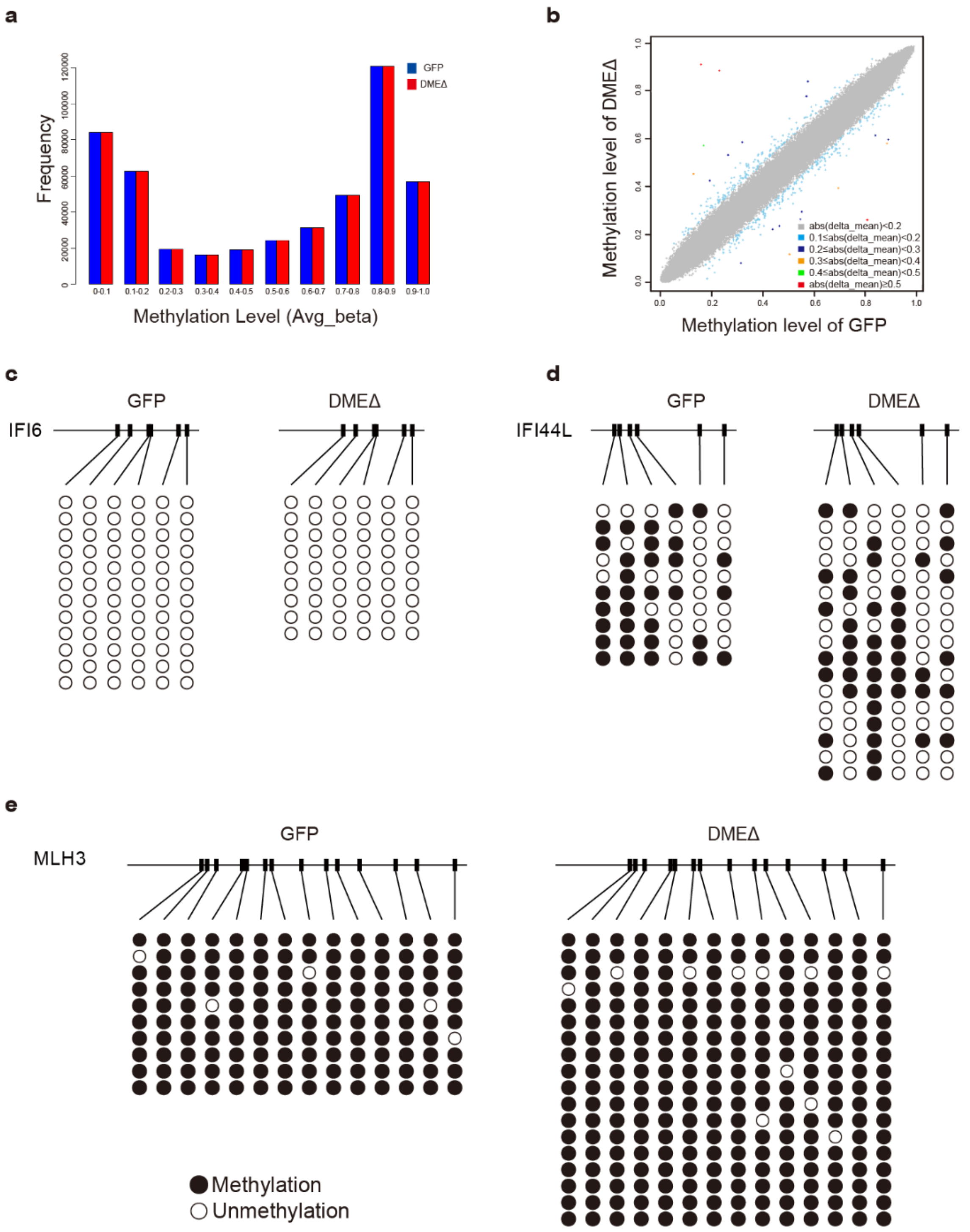

Supplementary Figure 5. DNA methylation analysis of 293T-GFP and 293T-DMEΔ cells.

Frequencies of the genes with different DNA methylation levels **(a)**, and scatter plot analysis of global DNA methylation levels **(b)** in the 293T-GFP versus the 293T-DMEΔ cells obtained from

an Infinium Human Methylation 450K BeadChip platform. Bisulfite sequencing analysis on the promoter regions of IFI6 (-301 ~ +9) **(c)**, IFI44L (-485 ~ +23) **(d)**, and MLH3 (-775 ~ -387) **(e)** as representative hypo-, intermediate, and hypermethylated genes, respectively. Closed circle, methylated cytosine; open circle, unmethylated cytosine. Samples were collected and analyzed 48 h after transfection.

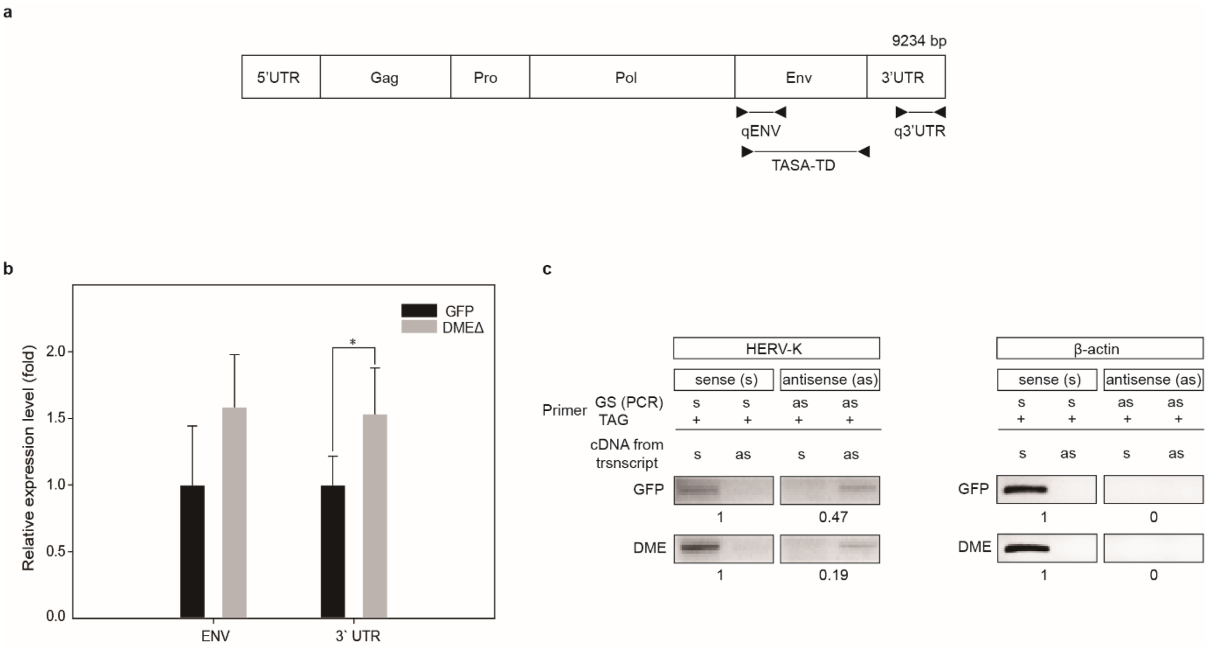

Supplementary Figure 6. Human ERV-K expression in 293T-GFP and 293T-DMEΔ cells.

(a) Schematic representation of human ERV-K gene. Arrowheads indicate the locations of primers for qRT-PCR and TASA-TD PCR. **(b)** Relative expression levels of ENV and 3' UTR of ERV-K in 293T-GFP and 293T-DMEΔ cells analysed by qRT-PCR. **(c)** Bidirectional transcription of ERV-K in 293T-GFP and 293T-DMEΔ cells analysed by TASA-TD PCR. The β-actin gene was used as a control for sense-only transcription. The PCR products were run on the same gel. Error bars represent mean ±S.D. of three independent experiments. *$p < 0.05$; paired sample t-test.

Supplementary Table 1. List of the oligonucleotides used for PCR cloning.

Name	Sequence (5` to 3`)	Note
DG49	aattgtcgactacaaaggagatggtgcac	*Sal* I site underlined
DG65	aattggatccttaggttttgttgttcttc	*Bam*H I site underlined
DG104	aattagatctccaaagaaaaagcgaaaggtaggtgtcgacaatt	*Bgl* II, *Sal* I sites underlined
DG105	aattgtcgacacctacctttcgctttttctttggagatctaatt	*Sal* I, *Bgl* II sites underlined
DG392	aattgctagccgccaccatggtgagcaaggg	*Nhe* I site underlined
DG393	aattgtttaaacttatctagatccggtggatc	*Pme* I site underlined
DG394	aattgtttaaacttaggttttgttgttcttc	*Pme* I site underlined
DG2106	cctccggtctacagctcccagcgtgag	Line 1 probe forward
DG2107	ccacttgaggaggcagtctgccc	Line 1 probe reverse

Supplementary Table 2. List of the primers for qRT-PCR analysis.

Gene	Forward sequence (5` to 3`)	Reverse sequence (5` to 3`)
Cyclin D1	aactacctggaccgcttcct	ccacttgagcttgttcacca
Cyclin B1	ataaagcttgtggccccttt	tcaattggatccccaggtaa
Cyclin A2	ctggtggtctgtgttctgtga	tcttggatgccagtcttactca
Cyclin E1	ccatcatgccgagggagc	ggtcacgtttgccttcctct
p21	atgaaattcacccccttcc	aggtgaggggactccaaagt
CDK1	ggtcaagtggtagccatgaaa	ccaggagggatagaatccaa
c-Myc	tcaagaggcgaacacacaac	ggccttttcattgttttcca
Rig-1	gttgtccccatgctgttctt	gcaagtcttacatggcagca
MDA5	accaaatacaggagccatgc	gcgatttccttcttttgcag
IFIT1	tctgcaaagtcccttttgct	ggggtgctctgtggtaagaa
IFIT2	tgctgaaagggagctgaaat	gacccagcaattcaggtgtt
IFIT3	gggcagactctcagatgctc	cagttgtgtccacccttcct
IFI6	cagcagcgtcgtcataggta	ggccaagaaggaagaagagg
IFITM2	ccgtgaagtctagggacagg	tgggatgatgatgagcagaa
IFI44L	tgttggcaaaagtgaagcaa	gggtccagttccaaatctga
CCR6	ggctgcaaatttgggtaaaa	cacaggagaagcctgaggac
Fas	tgtcatgaacccatgtttgc	gctgccatttgtagcaggtt
MLH3	ttgcattcattagcgtctgc	agggcctgttcttcaggatt
HSPA6	aggagatctcgtccatggtg	cgctgcgagtcattgaaata
HSPA1A	aggccaacaagatcaccatc	tcgtcctccgctttgtactt
DNAJB1	ttccccagacatcaagaacc	ccctctcatggtccacaact
HSPA2	aaaggtcgtctgagcaagga	ataggactccagggcgtttt
HSPB1	gagactgccgccaagtaaag	tttgacaggtggttgctttg
HSPH1	cacagccccaggtacaaact	tttgctttgtcagcatctgg
ISG15	caccgtgttcatgaatctgc	ctttatttccggcccttgat
OAS3	gtcaaacccaagccacaagt	tgtaggcacacctggtggta
CDC25C	gaacaggccaagactgaagc	gcccctggttagaatcttcc
IFN β	cattacctgaaggccaagga	cagcatctgctggttgaaga
IRF7	gcctggccaccataaaagc	gcgcacacatgaagtcacag
GAPDH	gagtcaacggatttggtcgt	gacaagcttcccgttctcag

Supplementary Table 3. List of the primers for bisulfite sequencing analysis.

Gene	Forward sequence (5` to 3`)	Reverse sequence (5` to 3`)
IFI44L	aaaatttaatttaattaaaaatttgtaagg	accaaacctatctacactaaaac
IFI6	agggggagttggtgattaggttttattaa	ctaactttttatcatcacttataaatcc
MLH3	ggggagatttaagttagtgaagagag	aaactatctaactcaactctttt

Supplementary Table S4. List of the primers for TASA-TD PCR analysis

Name	Sequence (5` to 3`)
Line 1 forward TAG	gcacacgacgacagacgacgcacccacttgaggaggcagtctgccc
Line 1 reverse TAG	gcacacgacgacagacgacgcaccctccggtctacagctcccagcgtgag
Line 1 forward	cctccggtctacagctcccagcgtgag
Line 1 reverse	ccacttgaggaggcagtctgccc
HERV-K forward TAG	gcacacgacgacagacgacgcacacaaaaccgccatcgtcatc
HERV-K reverse TAG	acaaaaccgccatcgtcatccatggtaagcgggatgtcact
HERV-K forward	catggtaagcgggatgtcact
HERV-K reverse	acaaaaccgccatcgtcatc
β-actin forward TAG	gcacacgacgacagacgacgcaccaaacatgatctgggtcatcttctc
β-actin reverse TAG	gcacacgacgacagacgacgcacgctcgtcgtcgacaacggctccggca
ß-actin forward	gctcgtcgtcgacaacggctccggca
ß-actin reverse	caaacatgatctgggtcatcttctc
TAG	gcacacgacgacagacgacgcac
